# Modulation of adipocyte G-protein expression in cancer cachexia by a lipid-mobilizing factor (LMF)

**DOI:** 10.1054/bjoc.2001.1992

**Published:** 2001-09

**Authors:** B Islam-Ali, S Khan, S A Price, M J Tisdale

**Affiliations:** Pharmaceutical Sciences Research Institute, Aston University, Birmingham, B4 7ET, UK

**Keywords:** lipid-mobilizing factor, G-protein expression, lipolysis

## Abstract

Adipocytes isolated from cachectic mice bearing the MAC 16 tumour showed over a 3-fold increase in lipolytic response to both low concentrations of isoprenaline and a tumour-derived lipid mobilizing factor (LMF). This was reflected by an enhanced stimulation of adenylate cyclase in plasma membrane fractions of adipocytes in the presence of both factors. There was no up-regulation of adenylate cyclase in response to forskolin, suggesting that the effect arose from a change in receptor number or G-protein expression. Immunoblotting of adipocyte membranes from mice bearing the MAC16 tumour showed an increased expression of Gαs up to 10% weight loss and a reciprocal decrease in Gα. There was also an increased expression of Gαs and a decrease in Gα in adipose tissue from a patient with cancer-associated weight loss compared with a non-cachectic cancer patient. The changes in G-protein expression were also seen in adipose tissue of normal mice administered pure LMF as well as in 3T3L1 adipocytes in vitro. The changes in G-protein expression induced by LMF were attenuated by the polyunsaturated fatty acid, eicosapentaenoic acid (EPA). This suggests that this tumour-derived lipolytic factor acts to sensitize adipose tissue to lipolytic stimuli, and that this effect is attenuated by EPA, which is known to preserve adipose tissue in cancer cachexia. © 2001 Cancer Research Campaign


*British Journal of Cancer* (2001) **85**(5), 758–763
© 2001 Cancer Research Campaign
doi: 10.1054/ bjoc.2001.1992, available online at http://www.idealibrary.com on IDEAL

### Modulation of adipocyte G-protein expression in cancer cachexia by a lipid-mobilizing factor (LMF)

**B Islam-Ali, S Khan, SA Price and MJ Tisdale**

Pharmaceutical Sciences Research Institute, Aston University, Birmingham B4 7ET, UK

**Summary** Adipocytes isolated from cachectic mice bearing the MAC 16 tumour showed over a 3-fold increase in lipolytic response to both low concentrations of isoprenaline and a tumour-derived lipid mobilizing factor (LMF). This was reflected by an enhanced stimulation of adenylate cyclase in plasma membrane fractions of adipocytes in the presence of both factors. There was no up-regulation of adenylate cyclase in response to forskolin, suggesting that the effect arose from a change in receptor number or G-protein expression. Immunoblotting of adipocyte membranes from mice bearing the MAC16 tumour showed an increased expression of G$\alpha$s up to 10% weight loss and a reciprocal decrease in G$\alpha$. There was also an increased expression of G$\alpha$s and a decrease in G$\alpha$ in adipose tissue from a patient with cancer-associated weight loss compared with a non-cachectic cancer patient. The changes in G-protein expression were also seen in adipose tissue of normal mice administered pure LMF as well as in 3T3L1 adipocytes in vitro. The changes in G-protein expression induced by LMF were attenuated by the polyunsaturated fatty acid, eicosapentaenoic acid (EPA). This suggests that this tumour-derived lipolytic factor acts to sensitize adipose tissue to lipolytic stimuli, and that this effect is attenuated by EPA, which is known to preserve adipose tissue in cancer cachexia. © 2001 Cancer Research Campaign

**Keywords**: lipid-mobilizing factor; G-protein expression; lipolysis

Cancer cachexia is associated with a severe and often irreversible loss of total body fat stores, which in mice has been shown to occur before the onset of anorexia (Costa, 1963). This depletion of host lipid stores may occur due to either a reduced rate of fatty acid deposition or an increased rate of mobilization (Thompson et al, 1981). Rates of whole body lipolysis have been shown to be greater in cachectic cancer patients with squamous cell carcinoma of the oesophagus than in healthy controls (Klein and Wolfe, 1990), suggesting production by tumour or host tissues of regulatory molecules involved in fat catabolism.

We have recently isolated a lipid-mobilizing factor (LMF) from the urine of cachectic cancer patients and shown it to be homologous in amino acid sequence with the plasma protein Zn-$\alpha_2$-glycoprotein (Todorov et al, 1998). This material initiates lipolysis through stimulation of adenylate cyclase in a GTP-dependent process in a manner analogous to the lipolytic hormones (Hirai et al, 1998). The elevation of cyclic AMP would lead to an activation of hormone-sensitive lipase (HSL). Thompson et al (1993) observed a 2-fold elevation in serum triacylglycerol and fatty acid levels in cancer patients compared with normal controls, and a 2-fold increase in the relative level of mRNA for HSL in adipose tissue. There was no change in the relative level of the mRNA for lipoprotein lipase (LPL), suggesting that an increased rate of mobilization, rather than deposition, was most important for the depletion of lipid stores. In addition the results suggest that the increased lipolysis in adipose tissue in cachexia may be due to an up-regulation of the lipolytic response in addition to the production of lipolytic stimuli.

*Received 7 March 2001*
*Revised 11 June 2001*
*Accepted 12 June 2001*

*Correspondence to:* MJ Tisdale

The present report examines the sensitivity to lipolytic stimuli of adipose tissue from mice bearing an experimental model of cancer cachexia (MAC16), as well as alterations in adipocyte G-protein expression during the development of cachexia, both in mice and man, and the role of LMF in this process.

### MATERIALS AND METHODS

Pure strain male NMRI mice (20–25 g) were obtained from our own inbred colony, and fragments of the MAC16 adenocarcinoma were implanted into the flanks by means of a trocar. Tumours were removed when they weighed between 0.1 and 0.6 *g* and before weight loss exceeded 20% of the total body weight. All animal experiements meet UKCCCK guidelines and were approved by the Home Office. Polyclonal antisera to G$\alpha$i (AS/7) and G$\alpha$s (RM/1) were purchased from NEN Life Science Products, Boston, MA, USA. Affinity purified rabbit anti-rat antibody to HSL was kindly provided by Dr Cecilia Holm, Lund University, Sweden. This antibody cross reacts with mouse HSL. Nitrocellulose membranes were purchased from Hoefer Scientific Instuments, San Francisco, CA, USA. Urine from cachectic cancer patients was kindly provided by Dr A Moses, Department of Surgery, Edinburgh Royal Infirmary, UK.

### Lipolytic assay

A single cell suspension of white adipocytes were prepared from finely minced epididymal fat pads of male NMRI mice bearing the MAC16 tumour, using collagenase digestion (Beck and Tisdale, 1987). Samples to be assayed were incubated with $10^5$–$2 \times 10^5$ adipocytes (determined by means of a haemocytometer) for 2 h at 37˚C in 1 ml of Krebs-Ringer bicarbonate buffer, pH 7.2. The concentration of glycerol released was determined enzymatically by the method of Wieland (1974). Control samples containing adipocytes alone were analysed to determine the spontaneous glycerol release. LMF was expressed as μmol glycerol released/$10^5$ adipocytes/2 h.

### Purification of LMF

Material capable of inducing lipolysis in isolated murine epididymal adipocytes was purified either from the solid MAC16 tumour or from the urine of cachectic cancer patients as previously described (Todorov et al, 1998). The steps consisted of ion exchange and exclusion chromatography followed by a Resource™ Iso hydrophobic interaction column to give an overall purification of 4200. This yielded a single protein of molecular weight 43 000 on denaturing polyacrylamide gels (Todorov et al, 1998).

### Isolation of adipocyte plasma membranes

The protocol was based on that previously described by Belsham et al (1980). All procedures were carried out at 4˚C. Adipocytes were homogenized in 10 mM Tris HCl, pH 7.4, containing 0.25 M sucrose and 2 mM EGTA, by aspirating through a Swinny filter at least 10 times. The homogenate was then centrifuged at 300 ***g*** for 5 min. The supernatant was removed and centrifuged at 30 000 ***g*** for 1 h. Plasma membranes were separated from other organelle membranes in the pellet on a self-forming Percoll gradient. The constituents were 0.25 M sucrose, 2 mM EGTA in 10 mM Tris. HCl, pH 7.4, Percoll and 2 M sucrose, 8 mM EGTA in 80 mM Tris. HCl, pH 7.4 mixed in a ratio of 32:7:1. The mixture was centrifuged at 10 000 ***g*** for 30 min and the membrane fractions were resuspended in 10 mM Tris. HCl, pH 7.4 containing 150 mM NaCl and 1 mM EGTA followed by centrifugation at 10 000 ***g*** for 2 min. The process was repeated twice. The washed plasma membranes were diluted in 10 mM Tris. HCl, pH 7.4 containing 0.25 M sucrose, 2 mM EGTA and 4 μM phenylmethylsulfonyl fluoride at 1–2 mg ml$^{-1}$ snap frozen in liquid nitrogen and stored at –70˚C until use.

### Adenylate cyclase assay

Adenylate cyclase activity in isolated plasma membranes was determined using the method of Salomon et al (1974). The incubation mixture contained 25 mM Tris. HCl, pH 7.4, 5 mM $MgCl_2$, 10 μM GTP, 8 mM creatine phosphate, 16 U ml$^{-1}$ creatine phosphokinase, 1 mM 3-isobutyl-1-methylxanthine, 1 mM cyclic AMP and 1 mM [α-$^{32}$P] ATP (sp. act. 30 μCi mmol$^{-1}$). The reaction was initiated by the addition of plasma membranes (typically 50 μg protein) to the assay mixture to give a total volume of 100 μl, which was incubated at 30˚C for 10 min. Reactions were terminated with 100 μl 2% sodium dodecyl sulfate (SDS) containing 40 mM ATP and 1.4 mM cyclic AMP. One microcurie of [$^3$H] cyclic AMP was added to each assay tube as an internal standard to monitor product recovery. Samples containing labelled nucleotides were diluted to 1 ml with water and loaded onto Dowex 50W8-400 columns primed with 10 ml of water. After washing twice with 1 ml of water the cyclic AMP was eluted with 3 ml of water into polypropylene tubes containing 200 μl of 1.5 M imidazole, pH 7.2. The samples were then applied to Alumina WN-3 columns (previously washed with 8 ml of 0.1 M imidazole, pH 7.5) and the eluate collected directly into scintillation vials containing Optiphase HiSafe 3 scintillation fluid. A further 1 ml of 0.1 M imidazole was added to the columns and the eluate was combined with the run through. The radioactivity was determined using a Tri-carb 2000A scintillation analyser.

### Western blot analysis

Samples of adipocyte plasma membrane (5 μg for Gs and 50 μg for Gi) were resolved on 10% sodium dodecylsulfate polyacrylamide gels and transferred to 0.45 μm nitrocellulose membranes, which had been blocked with 5% Marvel in Tris buffered saline, pH 7.5, at 4˚C overnight. The primary antibodies were used at a 1:2000 dilution, while the secondary antibody peroxidase conjugated monoclonal anti-rabbit immunoglobulin (Sigma Chemical Co, Dorset, UK) was used at a dilution of 1:1000. Incubation was for 2 h at room temperature and development was by enhanced chemiluminescence (ECL) (Amersham, UK). Blots were scanned by a densitometer to quantitate differences, and a parallel gel was silver stained to ensure equal loading. All blots were repeated at least 3 times on different incubations.

### 3T3L1 maintenance and differentiation

3T3 preadipocytes were maintained in Dulbecco's modified Eagle's medium (DMEM) containing 10% fetal calf serum under an atmosphere of 10% $CO_2$ in air. Cells were differentiated using a mixture of methylisobutylxanthine (0.5 mM), dexamethasone (0.25 μM) and insulin (1 μg ml$^{-1}$) according to the method of Frost and Lane (1985). The extent of differentiation to adipocytes was determined by staining for fat globules using Oil Red O. Cells were used for experimentation between 8 and 10 days after initiation of differentiation.

### RESULTS

Adipocytes isolated from the epididymal fat pads of male NMRI mice bearing the MAC 16 tumour and with 15% weight loss

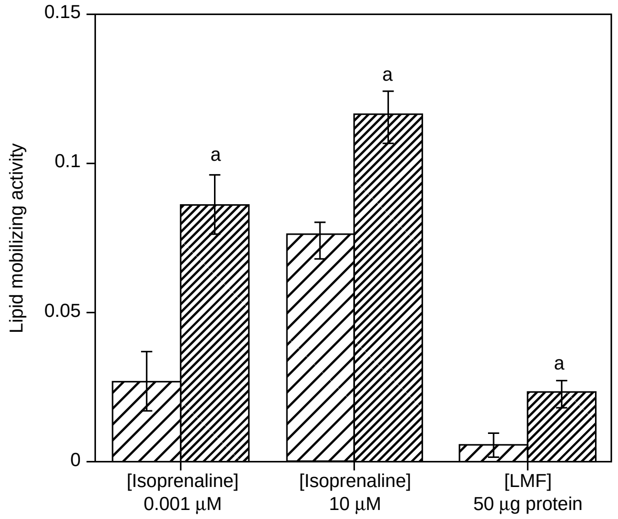

**Figure 1** Response of epididymal adipocytes from normal (hatched boxes) and cachectic (15% weight loss) (stippled boxes) male NMRI mice to lipolytic stimulation by isoprenaline and LMF. Basal lipolytic activity was not significantly different between adipocytes isolated from normal and cachectic mice (0.07 ± 0.004 and 0.057 ± 0.007 μmoles glycerol/$10^5$ adipocytes/2 h, respectively). Basal activity has been substracted from each of the values and the results are expressed as mean ± SEM where $n = 6$. Differences from normal adipocytes were determined by Student's *t*-test and are indicated as a $P \leq 0.05$

© 2001 Cancer Research Campaign 

BJOC 01-1992 758-763 20/8/01 3:55 pm Page 760

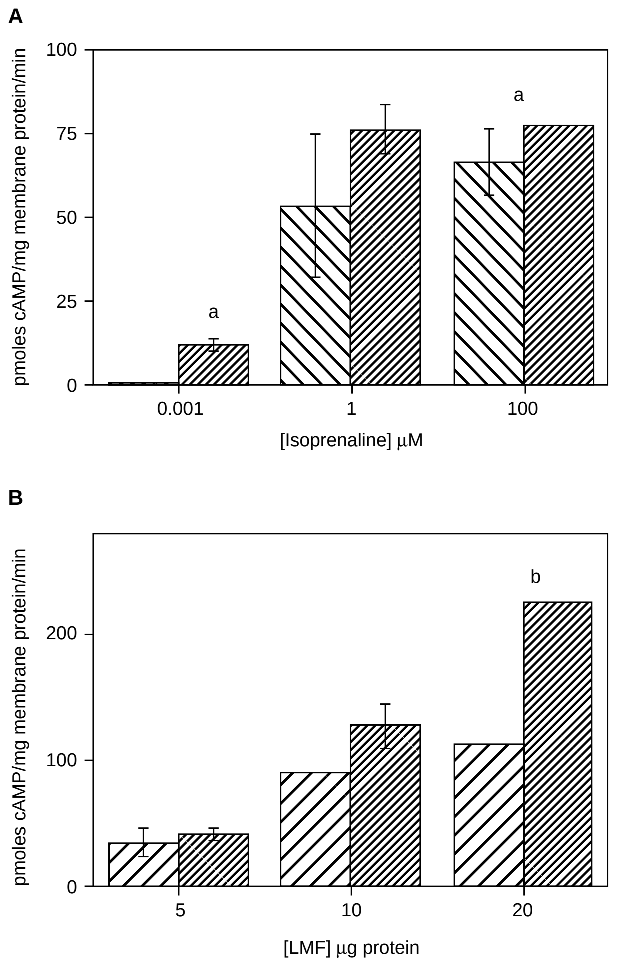

**Figure 2** Formation of cyclic AMP by white adipocyte membrane preparations (25 µg/incubation) in response to isoprenaline (**A**) or LMF (**B**). Adipocyte membranes were prepared from epididymal adipose tissue isolated either from normal male NMRI mice (hatched boxes) or from NMRI mice bearing the MAC16 tumour and with cachexia (15% weight loss) (stippled boxes). Basal activity was subtracted from the values, which are expressed as mean ± SEM, where $n = 4$ and represents 3 separate experiments. Error bars smaller than the width of the symbols are not shown. Differences from basal values were determined by Student's *t*-test and are indicated as **A**. $P \le 0.05$ and **B**. $P \le 0.01$

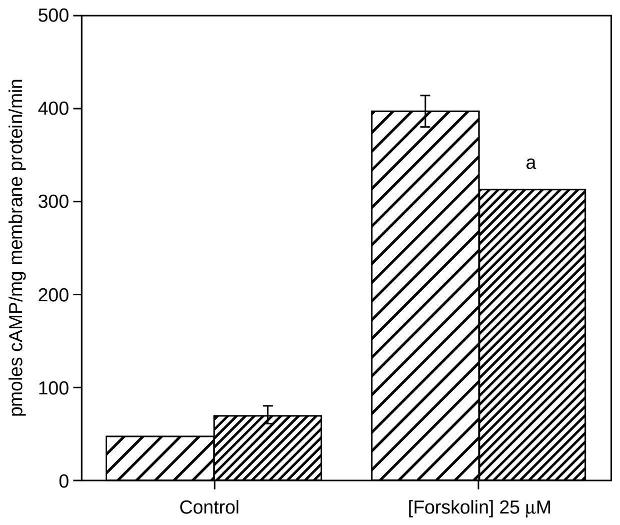

**Figure 3** Forskolin (25 µM) stimulation of adenylate cyclase in adipocyte plasma membranes (30 µg/incubation) isolated either from normal male NMRI mice (hatched boxes) or from NMRI mice bearing the MAC16 tumour and with cachexia (stippled boxes). Results are expressed as mean ± SEM where $n = 3$ and differences from normal adipocytes as determined by Student's *t*-test are indicated as a. $P \le 0.05$

showed over a 3-fold increase in response to lipolytic stimulation by both isoprenaline and LMF (Figure 1). The effect was more marked with low concentrations of isoprenaline. Plasma membranes prepared from adipocytes from mice bearing the MAC 16 tumour showed an enhanced stimulation of adenylate cyclase in the presence of isoprenaline (Figure 2A) or LMF (Figure 2B) when compared with adipocyte membranes from non-tumour bearing mice. The effect on isoprenaline stimulation was marked at submaximal concentrations (0.001 µM), where little or no response was observed in normal membranes, while there was no difference in response between adipocyte membranes prepared from cachectic and non-cachectic mice at optimal stimulatory concentrations (Figure 2A). The difference between adipocyte plasma membranes and intact cells may reflect differing GTP requirements for the adipocytes from cachectic mice. The activity of the LMF was potentiated in adipocyte membranes from cachectic mice in a dose-dependent manner (Figure 2B). Up-regulation of the response may be associated with the number or activity of receptors, G proteins or the cyclase catalytic element. The effect on the latter was determined by the use of forskolin, which acts directly to stimulate adenylate cyclase. There was no up-regulation of response to forskolin in adipocyte membranes from cachectic mice (Figure 3), and there appeared to be greater cyclic AMP production using plasma membranes from non-cachectic animals.

Immunoblotting of plasma membranes with the specific Gαs antibody showed a marked increase in both 46 and 54 kDa Gαs splice variants with increasing weight loss up to 10% (Figure 4A). Thereafter, the levels of both variants decreased to that found in mice with 5% weight loss, but were still significantly increased above that found in mice with no weight loss. In contrast there was a decrease in the intensity of the 40 kDa band detected with anti-Gαi with increasing weight loss reaching a minimum at 10% weight loss (Figure 4B) and thereafter increased with additional host wasting. The ratio of Gαs to Gαi detected in adipocyte plasma membranes increased markedly with increasing weight loss (Table 1) reaching a maximum at 10% weight loss, whereafter the value decreased, but still remained elevated over that found in mice with no weight loss up to 15% weight loss. Treatment of mice with EPA (0.5 g $kg^{-1}$) which completely attenuated weight loss (Beck et al, 1991) returned the Gαs/Gαi ratio back to control levels (Table 1).

**Table 1** Gαs/Gαi ratio in adipocyte plasma membranes from mice bearing the MAC 16 tumour or administered LMF[a]

| **Weight loss or treatment** | **Gαs/Gαi** |
|---|---|
| 0 | 0.83 |
| 5 | 1.72 |
| 10 | 3.14 |
| 15 | 2.18 |
| 20 | 0.73 |
| LMF | 2.23 |
| MAC 16 + EPA | 0.66 |

[a]Determined from densitometric analysis of the blot shown in Figure 4.

 *© 2001 Cancer Research Campaign*

BJOC 01-1992 758-763 20/8/01 3:55 pm Page 761

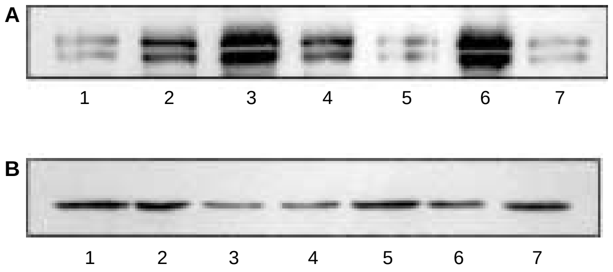

**Figure 4** Immunodetection of Gαs (**A**) and Gαi (**B**) in white adipocyte plasma membranes isolated from mice bearing the MAC16 tumour with 0% (lane 1), 5% (lane 2), 10% (lane 3), 15% (lane 4) and 20% (lane 5) weight loss. Mice were also administered LMF (8 μg b.d.) for 48 h which produced 7% weight loss and animals were terminated 12 h after the last injection and adipocyte membranes were blotted for Gαs and Gαi (lane 6), while mice bearing the MAC16 tumour were treated with EPA (0.5 g $kg^{-1}$) (lane 7). Band volume was measured as an indication of the quantity of G-protein present

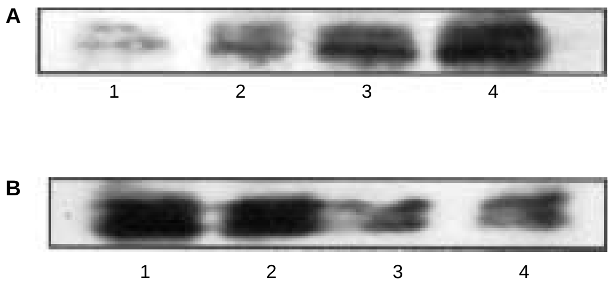

**Figure 5** Immunodetection of Gαs (**A**) and Gαi (**B**) in subcutaneous (lanes 1 and 3) and omental (lanes 2 and 4) adipose tissue from a cancer patient with no weight loss (lanes 1 and 2) and from a cachectic subject with 15% weight loss (lanes 3 and 4)

A similar change in G-protein expression was found in adipose tissue of a patient with cancer cachexia, when compared with a non-cachectic cancer patient, both with colonic cancer. Thus there was an enhanced expression of Gαs in both omental and subcutaneous adipose tissue in the cachectic subject (Figure 5A), while expression of Gαi was markedly reduced (Figure 5B). Thus the Gαs/Gαi ratios were increased to a comparable extent as that found in mice bearing the MAC16 tumour (Table 2).

To determine if changes in G-protein expression in adipose tissue were due to LMF ex-breeder male mice were injected i.v. with purified human LMF (8 μg b.d. for 48 h). Plasma membrances isolated from the epididymal fat pads of such animals showed an increase in expression of Gαs, while Gαi remained unchanged (Figure 4) raising the Gαs/Gαi ratio to levels found in MAC16 mice with 15% weight loss (Table 1). Neither LMF (Hirai et al, 1998) nor the MAC16 tumour (Beck and Tisdale, 1987) induce anorexia, so the effect was not due to fasting. To investigate whether this was a direct effect of LMF on G-protein expression 3T3L1 adipocytes were treated with increasing concentrations of LMF from 12 to 580 nM for 24 h and plasma membranes were isolated and subjected to immunoblotting for Gαs and Gαi (Figure 6). There was an increase in expression of Gαs with increasing concentrations of LMF (Figure 6A), which was not seen in 3T3L1 cells pretreated with 50 μM EPA for 2 h prior to the addition of LMF (Figure 6B). There was a decrease in Gαi expression with increasing concentrations of LMF with the maximum decrease at 580 nM LMF (Figure 6C). In 3T3 cells pretreated with 50 μM EPA there was no down-regulation of Gαi expression with increasing concentrations of LMF (Figure 6D). The Gαs:Gαi

**Table 2** Gαs/Gαi ratio in subcutaneous (s.c.) and omental (om) adipocyte plasma membranes from cachectic and non-cachectic subjects[a]

| Subject | Gαs/Gαi |
|---|---|
| Non-cachectic (s.c.) | 0.32 |
| Cachectic (s.c.) | 1.17 |
| Non-cachectic (om) | 0.53 |
| Cachectic (om) | 1.29 |

[a]Determined from densitometric analysis of the blot shown in Figure 5.

**Table 3** Gαs/Gαi ratio in adiopocyte membranes from 3T3L1 cells in the absence (A) or presence (B) of 50 μM EPA

| | Gαs/Gαi[a] | |
|---|---|---|
| **Concentration of LMF (nM)** | **A** | **B** |
| 0 | 1.00 | 1.00 |
| 12 | 1.58 | 0.94 |
| 58 | 1.46 | 0.94 |
| 174 | 2.25 | 1.08 |
| 406 | 2.85 | 1.23 |
| 580 | 2.62 | 1.26 |

[a]Normalized to control value of 1.00.

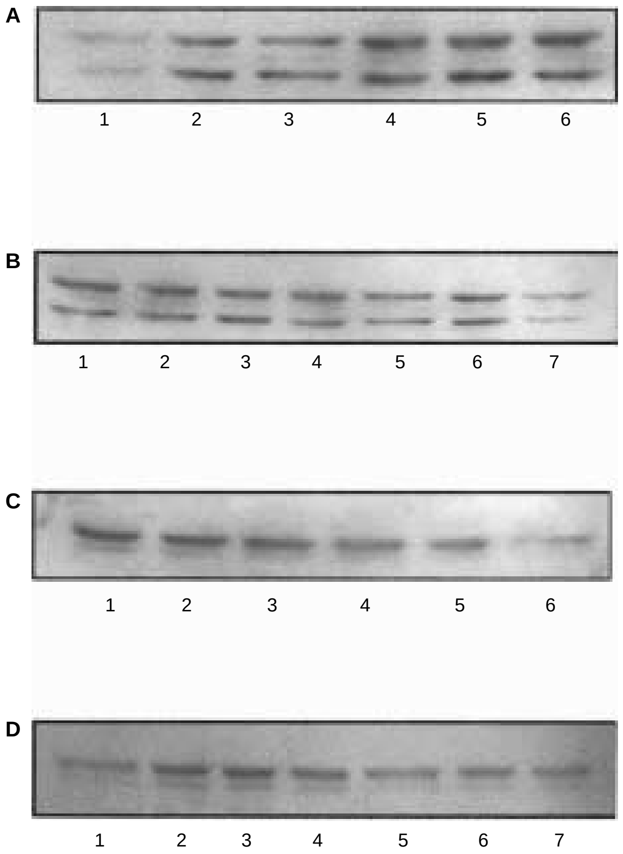

**Figure 6** Immunodetection of Gαs (**A** and **B**) and Gαi (**C** and **D**) in 3T3L1 adipocytes incubated either alone (lane 1) or with 12 nM (lane 2); 58 nM (lane 3); 174 nM (lane 4); 406 nM (lane 5) and 580 nM (lane 6) LMF for 24 h in either the absence (**A** or **C**) or with a 2 h pretreatment with 50 μM EPA (**B** and **D**)

*© 2001 Cancer Research Campaign* 

BJOC 01-1992 758-763 20/8/01 3:55 pm Page 762

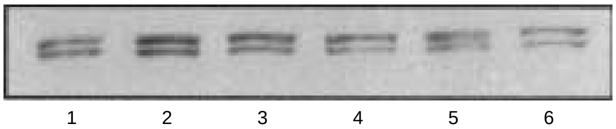

**Figure 7** Immunodetection of HSL in 3T3L1 adipocytes after incubation either alone (lane 1) or with 12 nM (lane 2); 58 nM (lane 3); 174 nM (lane 4); 406 nM (lane 5) and 580 nM (lane 6) LMF for 24 h. Monolayers were rinsed 3 times with ice-cold PBS, scraped into 0.25 M sucrose, 1 mM EDTA, 1 mM dithioerythritol, 10 μg $ml^{-1}$ leupeptin and 10 μg $ml^{-1}$ antipain and sonicated (3 × 10 s) on ice. The cell homogenate was centrifuged at 15 000 rpm for 15 min at 4°C and the supernatant was used for Western blotting as described in methods using 30 μg protein per lane. The primary antibody, rabbit anti-rat HSL was used at a dilution of 1:2000, while the secondary antibody, peroxidase conjugated donkey anti-rabbit immunoglobulin (Amersham, UK) was used at a dilution of 1:1000. Detection was by ECL. Densitometric analysis showed a significant ($P$ < 0.05 from control by Student's *t*-test) increase in expression of HSL with 12 nM (55% increase) and 58 nM (40% increase) LMF. The blot is a representative example from 3 separate experiments carried out on different occasions ratio of cells treated with LMF with and without EPA is shown in Table 3. Over 2-fold stimulation of Gαs:Gαi was seen at concentrations of LMF greater than 174 nM, while in cells pretreated with EPA the Gαs:Gαi ratio remained near unity at all concentrations of LMF. These results confirm the ability of LMF to directly alter levels of expression of Gαs and Gαi and provide an explanation for the inhibitory effect of EPA on this process.

Low concentrations of LMF (12–58 nM) also caused an increased expression of HSL in 3T3L1 adipocytes (Figure 7). Thus LMF not only stimulates adenylate cyclase in adipocytes (Price and Tisdale, 1998), but also modifies G-protein expression and HSL to maximally activate lipolysis.

### DISCUSSION

Both the induction of lipolysis and formation of cyclic AMP by both isoprenaline and LMF have been shown to be up-regulated in epididymal fat pads from cachectic mice bearing the MAC16 tumour compared with non-tumour-bearing mice. This suggests that a feature common to the pathways of both agents has been up-regulated. This appears not to be adenylate cyclase since forskolin, which by-passes receptor binding and activates adenylate cyclase directly, did not produce an enhanced response using adipocyte membranes from cachectic animals. This suggests that the up-regulated response in adipose tissue from cachectic animals is due to modification of receptor number of guanine nucleotide-binding protein (G-protein) expression.

The G-proteins involved in the adenylate cyclase pathway consist of Gs and Gi families that stimulate and inhibit adenylate cyclase, respectively (Levitzki, 1987). Adipocyte plasma membranes contain 2 Gs α-subunits and 2 Gi α-subunits, but not Go (Hinsch et al, 1988). A number of stimuli control G-protein expression in adipose tissue. Thus depletion of testosterone by castration has been shown to induce a down-regulation of both Gαs and Gαi 1 + 2, with testosterone replacement restoring Gαs expression (Dieudonne et al, 1993). A concurrent up-regulation of the expression of Gαi 1, Gαi 2 and Gαi 3, but not Gαs, has been noted in adipocytes isolated from hypothyroid rats (Milligan and Saggerson, 1990), indicating an increased efficiency of antilipolytic agents such as adenosine, and correlating with the gain in body weight often observed in individuals exhibiting hypothyroidism. Patients with Gs deficiency are obese and show a decreased lipolytic response to adrenaline (Carel et al, 1999). In addition heterologous desensitization of lipolysis appears at least partly due to down-regulation of Gi (Green et al, 1992). Stimulation of lipolysis by tumour necrosis factor-α (TNF-α) appears to be by Gi protein down-regulation blunting endogenous inhibition of lipolysis by adenosine (Gasic et al, 1999). Thus in addition to agonist-mediated receptor expression, adipocyte response to lipolytic stimuli also depends upon the expression and conformation of G-proteins.

To date there have been no measurements of G-protein expression in adipose tissue in cancer cachexia. This study reports alterations in adipocyte G-proteins with the development of the cachectic state in both mice bearing the cachexia-inducing MAC16 tumour and in a patient with cancer cachexia. The changes consisted principally of a reduction in membrane Gαi expression allied with increased expression of Gαs, changes which would favour mobilization of lipid stores from adipocytes and hence facilitate host tissue catabolism. Such alterations were observed in mice manifesting up to 10–15% loss of their original body weight and correlates with maximal production of LMF by the tumour (Groundwater et al, 1990).

Similar changes in G-protein expression were also seen in mice administered LMF, as well as in 3T3L1 adipocytes in vitro, showing that the observed changes in G-protein expression were a direct effect of LMF. LMF was also found to stimulate expression of HSL in 3T3 adipocytes, which may explain the increased levels observed in adipose tissue of cancer patients (Thompson et al, 1993).

The polyunsaturated fatty acid, eicosapentaenoic acid (EPA) has been shown to attenuate the development of cachexia in both the MAC16 model (Beck et al, 1991) and in patients with unresectable pancreatic cancer (Wigmore et al, 1996), with preservation of adipose stores. Using isolated white adipocytes we have previously shown EPA inhibition of both lipolysis and adenylate cyclase stimulation by LMF to be a pertussis toxin-sensitive process, suggesting the involvement of Gαi (Price and Tisdale, 1998). The effect appears to arise from a direct attenuation of the action of LMF on G-protein expression in adipose tissue, resulting in an increased Gαi/Gαs expression.

Thus tumours secreting LMF will maximize catabolism of adipose tissue by the continuous production of this lipolytic stimulus, together with increases in Gαs/Gαi and HSL, which will sensitize the adipocytes to a range of lipolytic stimuli. Such changes would explain the 85% fall in total body fat reported for lung cancer patients who had lost 30% of their pre-illness stable weight (Fearon, 1992) if energy utilization was also increased. In mice administered LMF a 3-fold increase in oxygen consumption by interscapular brown adipose tissue was observed (Hirai et al, 1998) suggesting an increase in thermogenesis. The mechanism by which this occurs will be the subject of further investigation.

### ACKNOWLEDGEMENTS

We thank Mr M Wynter and Mr W Fleary for the tumour transplantations and Professor K Fearon, Department of Surgery, Edinburgh Royal Infirmary, UK for the samples of adipose tissue from cancer patients. SAP gratefully acknowledges receipt of a research studentship for Scotia Pharmaceuticals Ltd.

### REFERENCES

Beck SA and Tisdale MJ (1987) Production of lipolytic and proteolytic factors by a murine tumor-producing cachexia in the host. *Cancer Res* **47**: 5919–5923

*© 2001 Cancer Research Campaign*

BJOC 01-1992 758-763 20/8/01 3:55 pm Page 763

Beck SA, Smith KL and Tisdale MJ (1991) Anticachectic and antitumor effect of eicosapentaenoic acid and its effect on protein turnover. *Cancer Res* **51**: 6089–6093

Belsham GJ, Denton RM and Tanner MJA (1980) Use of a novel rapid preparation of fat-cell plasma membranes employing percoll to investigate the effects of insulin and adrenaline on membrane protein phosphorylation within intact fat cells. *Biochem J* **192**: 457–467

Carel JC, Stunff CL, Condamine L and 5 others (1999) Resistance to the lipolytic action of epinephrine: A new feature of protein Gs deficiency. *J Clin Endocrinol Metab* **84**: 4127–4131

Costa G (1963) Cachexia, the metabolic component of neoplastic disease. *Progr Exp Tumour Res* **3**: 321–369

Dieudonne M-N, Pecquery R, Dausse J-P and Giudicelli Y (1993) Regulation of white adipocyte guanine nucleotide binding proteins Gsα and Gi$\alpha_{1-2}$ by testosterone in vivo: influence of regional fat distribution. *Biochim Biophys Acta* **1176**: 123–127

Gasic S, Tian B and Green A (1999) Tumor necrosis factor α stimulates lipolysis in adipocytes by decreasing Gi protein concentrations. *J Biol Chem* **274**: 6770–6775

Green A, Milligan G and Dobias SB (1992) Gi Down-regulation as a mechanism for heterologous desensitization in adipocytes. *J Biol Chem* **267**: 3223–3229

Groundwater P, Beck SA, Barton C, Adamson C, Ferrier IN and Tisdale MJ (1990) Alteration of serum and urinary lipolytic activity with weight loss in cachectic cancer patients. *Br J Cancer* **62**: 816–821

Hinsch KD, Rosenthal W, Spicher K, Binder T, Gauespohl H, Frank R, Scultz G and Joost HG (1988) Adipose plasma membranes contain two Gi subtypes but are devoid of Go. *FEBS Lett* **238**: 191–196

Hirai K, Hussey HJ, Barber MD, Price SA and Tisdale MJ (1998) Biological evaluation of a lipid mobilizing factor (LMF) isolated from the urine of cancer patients. *Cancer Res* **58**: 2359–2365

Klein S and Wolfe RR (1990) Whole body lipolysis and triglyceride-fatty acid cycling in cachectic patients with oesophageal cancer. *J Clin Invest* **86**: 1403–1408

Levitzki A (1987) Regulation of adenylate cyclase by hormones and G-proteins. *Fed Eur Biochem Soc Lett* **211**: 113–118

Milligan G and Saggerson ED (1990) Concurrent up-regulation of guanine-nucleotide-binding proteins $G_i1\alpha$, $G_i2\alpha$ and $G_i3\alpha$ in adipocytes of hypothyroid rats. *Biochem J* **270**: 765–769

Price SA and Tisdale MJ (1998) Mechanism of inhibition of a tumor lipid-mobilizing factor by eicosapentaenoic acid. *Cancer Res* **58**: 4827–4831

Salomon Y, Londos C and Rodbell M (1974) A highly sensitive adenylate cyclase assay. *Anal Biochem* **58**: 541–548

Thompson MP, Koons JE, Tan ETH and Grigor MR (1981) Modified lipoprotein lipase activities, rates of lipogenesis and lipolysis as factors leading to lipid depletion in C57BL mice bearing the preputial gland tumor, ESR-586. *Cancer Res* **41**: 3228–3232

Thompson MP, Cooper ST, Parry BR and Tuckey JA (1993) Increased expression of the mRNA for the hormone-sensitive lipase in adipose tissue of cancer patients. *Biochim Biophys Acta* **1180**: 236–241

Todorov PT, McDevitt TM, Meyer DJ, Ueyama H, Ohkubo I and Tisdale MJ (1998) Purification and characterization of a tumor lipid mobilizing factor (LMF). *Cancer Res* **58**: 2353–2358

Wieland O (1974) Glycerol UV method. In *Methods of Enzymatic Analysis.* 4. Bergmeyer HU (ed) pp 1404–1409, Academic Press: London

Wigmore SJ, Ross JA, Falconer JS, Plester CE, Tisdale MJ, Carter DC and Fearon KCH (1996) The effect of polyunsaturated fatty acids on the progress of cachexia in patients with pancreatic cancer. *Nutrition* **12**(Suppl): S27–S30

*© 2001 Cancer Research Campaign*
